# Predicting the Feasibility of Curative Resection in Low Rectal Cancer: Insights from a Prospective Observational Study on Preoperative Magnetic Resonance Imaging Accuracy

**DOI:** 10.3390/medicina60020330

**Published:** 2024-02-15

**Authors:** Cristian-Constantin Volovat, Dragos-Viorel Scripcariu, Diana Boboc, Simona-Ruxandra Volovat, Ingrid-Andrada Vasilache, Corina Lupascu-Ursulescu, Liliana Gheorghe, Luiza-Maria Baean, Constantin Volovat, Viorel Scripcariu

**Affiliations:** 1 Department of Radiology, “Grigore T. Popa” University of Medicine and Pharmacy, 700115 Iasi, Romaniacorina.ursulescu@gmail.com (C.L.-U.); lgheorghe123@gmail.com (L.G.);; 2 Department of Surgery, “Grigore T. Popa” University of Medicine and Pharmacy, 700115 Iasi, Romania; 3 Department of Medical Oncology-Radiotherapy, “Grigore T. Popa” University of Medicine and Pharmacy, 700115 Iasi, Romaniacvolovat@gmail.com (C.V.); 4 Department of Mother and Child Care, “Grigore T. Popa” University of Medicine and Pharmacy, 700115 Iasi, Romania

**Keywords:** rectal adenocarcinoma, magnetic resonance imaging, predictive performance, combined models

## Abstract

*Background and Objectives*: A positive pathological circumferential resection margin is a key prognostic factor in rectal cancer surgery. The point of this prospective study was to see how well different MRI parameters could predict a positive pathological circumferential resection margin (pCRM) in people who had been diagnosed with rectal adenocarcinoma, either on their own or when used together. *Materials and Methods*: Between November 2019 and February 2023, a total of 112 patients were enrolled in this prospective study and followed up for a 36-month period. MRI predictors such as circumferential resection margin (mCRM), presence of extramural venous invasion (mrEMVI), tumor location, and the distance between the tumor and anal verge, taken individually or combined, were evaluated with univariate and sensitivity analyses. Survival estimates in relation to a pCRM status were also determined using Kaplan–Meier analysis. *Results*: When individually evaluated, the best MRI predictor for the detection of a pCRM in the postsurgical histopathological examination is mrEMVI, which achieved a sensitivity (Se) of 77.78%, a specificity (Sp) of 87.38%, a negative predictive value (NPV) of 97.83%, and an accuracy of 86.61%. Also, the best predictive performance was achieved by a model that comprised all MRI predictors (mCRM+ mrEMVI+ anterior location+ < 4 cm from the anal verge), with an Se of 66.67%, an Sp of 88.46%, an NPV of 96.84%, and an accuracy of 86.73%. The survival rates were significantly higher in the pCRM-negative group (*p* < 0.001). *Conclusions*: The use of selective individual imaging predictors or combined models could be useful for the prediction of positive pCRM and risk stratification for local recurrence or distant metastasis.

## 1. Introduction

There are about 46,000 new cases of rectal cancer diagnosed each year in the United States [1]. Most of these are adenocarcinomas [2]. Similar epidemiological data were retrieved from Europe, and recent GLOBOCAN statistics for 2022 indicated that colorectal cancer was the third most frequently diagnosed type of cancer in males, with 92,942 newly diagnosed cases in Eastern Europe [3]. The same data registry outlined colorectal cancer as the second most frequently diagnosed type of tumor in the female population in Eastern Europe, with 86,575 new cases diagnosed in 2022. Romania closely followed this epidemiological pattern of colorectal cancer, and in 2022, 8056 new cases were diagnosed in males (the third most frequent type of cancer) and 5485 new cases were diagnosed in females (the second most frequent type of cancer) [3]. The treatment approach for these cases aligns with that for anal cancer, involving initial chemoradiotherapy (CRT) instead of surgery. CRT entails the use of radiotherapy alongside concurrent chemotherapy based on fluoropyrimidine [4].

The objective of the pretreatment staging evaluation is to determine the existence of distant metastatic disease and ascertain the tumor location in the rectum and its local extent [5]. Prior to treatment, it is crucial to make a precise evaluation of the tumor’s location and extent in order to determine the most suitable surgical approach and identify patients who are eligible for initial therapy. This may involve options such as long-course chemoradiotherapy (CRT), short-course radiation therapy (RT) alone, or a combination of short-course radiation followed by chemotherapy or neoadjuvant chemotherapy and CRT before proceeding with surgery [6,7].

Pelvic magnetic resonance imaging (MRI) is the recommended imaging technique for assessing the primary tumor’s extent [8]. It can offer valuable insights into the circumferential resection margin (CRM), potential organ and structure invasion, and involvement of pelvic sidewall lymph nodes [9]. When it comes to assessing rectal cancers, MRI outperforms CT scans in determining the extent of invasion, whether adjacent structures are affected, and the presence of perirectal nodal involvement [10].

The MERCURY II study has demonstrated the effectiveness of MRI in evaluating the relationship between tumors and the mesorectal fascia [11]. These findings can be used to predict outcomes for lower rectal tumors in terms of pathological (positive) circumferential resection margin (pCRM). The study found that the presence of extramural venous invasion on MRI (mrEMVI), tumors located less than 4 cm from the anal verge, and tumors located in the anterior region are all factors that independently increase the risk of pCRM [11].

In a recent study conducted by Poulsen et al., the impact of tumor height on the accuracy of preoperative MRI-based staging in patients with rectal adenocarcinoma was examined [12]. The researchers found that tumor height did not affect the ability of preoperative MRI to accurately stage rectal tumors, as confirmed by postoperative histopathological assessments. In the same study, patients who had undergone neoadjuvant CRT experienced MRI overstaging of low rectal adenocarcinoma as a result of post-radiation fibrosis. The authors also discovered that the extramural tumor depth (EMD) identified through MRI examination accurately predicted the EMD determined through histopathological evaluation.

To achieve a successful curative resection, it is important to perform a thorough removal of the cancer by ensuring negative margins on a histological level [13]. Additionally, a total mesorectal excision (TME) should be carried out, which involves removing local lymph nodes through transabdominal procedures such as low anterior resection (LAR) or abdominoperineal resection (APR) [14,15]. These surgical techniques can be performed via laparotomy, laparoscopy, or robotic approaches, the last two approaches having the advantages of relatively short recovery time and lower postoperative complication rates [16].

There are several predictors of a positive pCRM cited in the literature. These include clinical, intraoperative, or imaging risk factors. In a multicenter randomized phase III trial (ACCORD12/0405 PRODIGE 2), the authors identified abdominoperineal resection, vascular tumor invasion, and poor histological response (modified Dworak 0–2) as independent predictors for positive pCRM [17].

Moreover, the type of facility where the surgery is performed, the age and performance status of the patients, the number of harvested lymph nodes, clinical T and N stage, histologic type, and tumor size and grade were also cited as important predictors for the positivity of pCRM [18,19,20].

The aim of this prospective study was to evaluate the predictive performance of various MRI parameters individually or combined for the prediction of pCRM in patients diagnosed with rectal adenocarcinoma.

## 2. Materials and Methods

This prospective observational study was conducted in the first Oncologic Surgical Clinic from the Regional Institute of Oncology, Iasi, Romania, between November 2019 and February 2023. Ethical approval for this study was obtained from the Institutional Ethics Committees of the University of Medicine and Pharmacy ‘Grigore T. Popa’ (No. 23103/23 October 2019) and of the Regional Institute of Oncology (No. 245/3 July 2019).

In this study, we included patients diagnosed with low rectal tumors (less than 8 cm from the anal verge), a histopathological examination that indicated adenocarcinoma type, who had a preoperative pelvic MRI for staging, and who received neoadjuvant therapy, as well as those who offered their informed consent for participating in this study.

The exclusion criteria comprised patients with rectal cancers who needed emergency surgery, other subtypes of rectal cancer, loss of follow-up, incomplete medical data, or lack of informed consent.

The following data were recorded: demographic and clinical characteristics, preoperative MRI parameters, histopathological examination of preoperative biopsies and postoperative specimens, type of surgery, postsurgical evolution, and survival. All patients underwent pelvic MRI examination using the SIEMENS MAGNETOM Avanto I-class 1.5 Tesla machine (Siemens Healthcare GmbH, Erlangen, Germany).

The examination protocol included the visualization of the pelvis in all three planes:

− Sagittal—this plane is used to locate the tumor and to plan the axial and coronal sequences;
− Axial—the plane is angled perpendicular to the tumor to correctly visualize the extension of the tumor against the rectal wall, as well as the distance between the tumor and the mesorectal fascia (MRF);
− Coronal—the plane is angled parallel to the axis of the tumor, which is perpendicular to the axial series.

**Figure 1:**
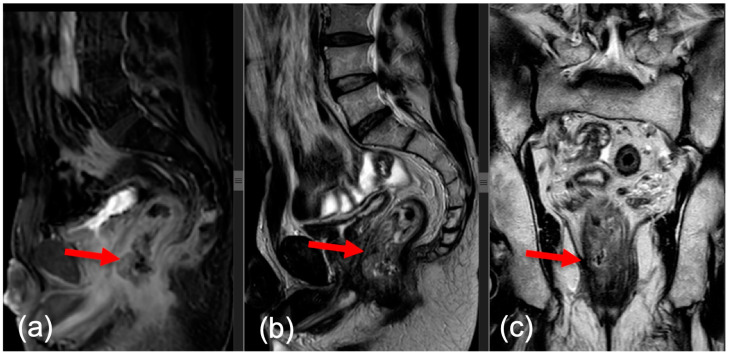
Post-contrast sagittal image (**a**), sagittal T2 image (**b**), and coronal T2 image (**c**). Expansive tumor of the lower rectus (red arrow) with invasion in the muscularis propria and with the retraction of the colic wall.

**Figure 2:**
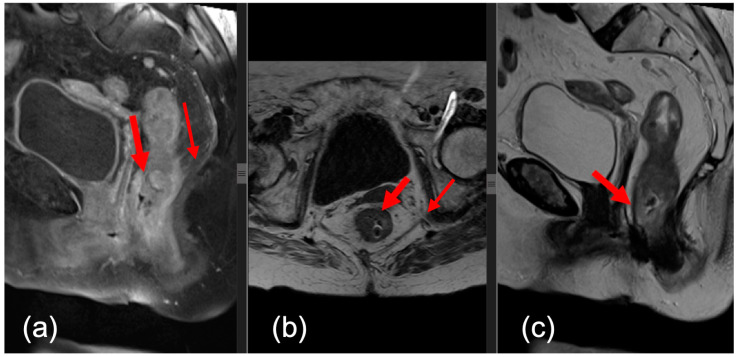
Post-contrast sagittal T1 image (**a**), axial T1 image (**b**), and sagittal T2 image (**c**). Expansive tumor of the lower rectus (red arrow) with extension towards the anal canal.

The sequences used were the following: multiplanar T2w and T1w, which provided valuable morphological information due to the high resolution of anatomical structures. The T2w sequence was ≤3 mm thick. The preoperative parameters included tumor size, location, distance from anal verge, extramural venous invasion (EMVI) status (tumor invasion into veins beyond muscularis propria), and MRI CRM (mCRM) positivity. Figure 1 and Figure 2 outline two MRI images of rectal tumor invasion.

After the elective surgical procedure, the intestinal specimens were evaluated by specialized pathologists in oncology from the Regional Oncologic Institute according to standard procedure. A positive pathological circumferential resection margin was considered a distance of less than 1 mm from the tumor cells to the cut specimen margin. The patients were segregated into 2 groups based on the presence or absence of a postsurgical positive circumferential resection margin: group 1 (positive, n = 9 patients) and group 2 (negative, n = 103 patients).

In the first phase of our analysis, we used descriptive statistics and a comparison of categorical variables (Pearson’s χ2 test) or continuous variables (Student *t*-test) between groups.

In the second stage of the analysis, we used a generalized linear model (GLM) to identify MRI predictors for a positive circumferential resection margin after surgery and quantified their impact as risk ratio (RR).

In the third stage of the analysis, we performed a sensitivity analysis for the quantification of MRI predictors’ performance.

Finally, we provided a survival analysis of our cohort of patients using Kaplan–Meier estimates, segregated depending on the presence of a positive pathological circumferential resection margin and adjusted for the age covariate.

A *p*-value less than 0.05 was considered statistically significant. These analyses were performed using STATA SE (version 17, 2023, StataCorp LLC, College Station, TX, USA).

## 3. Results

A total of 112 patients were included in the analysis, and their clinical and paraclinical characteristics are presented in Table 1. The mean age and standard deviation were significantly higher for the group of patients with positive circumferential resection margin determined in histopathological examination (68.61 ± 9.45 versus 61.33 ± 12.30, *p* = 0.03). Other patients’ characteristics such as gender, medium of living, body mass index (BMI), or smoking status did not significantly differ between groups (*p* > 0.05).

The first group of patients presented with significantly higher rates of positive mCRM compared to the second group (66.67% versus 19.42%, *p* = 0.001). Moreover, this group also presented with significantly higher rates of EMVI on MRI examination compared to the second group (77.78% versus 12.62%, *p* < 0.001).

Regarding the tumor location and size, only anteriorly located tumors were significantly more frequently encountered in the first group (*p* = 0.01), while the distance from the anal verge of the tumors did not significantly differ between groups (*p* = 0.32).

In the second stage of our analysis, we evaluated a generalized linear model that included MRI predictors (mCRM, mrENVI, distance from the anal verge, and tumor location) and an outcome represented by the presence of pCRM. The impact of predictor variables on the outcome was quantified by risk ratios (RRs) along with confidence intervals (CIs) that are presented in Table 2. Our results indicated that a positive mrEMVI had the highest impact on the discovery of a pCRM on pathological examinations (RR: 40.97, 95%CI: 10.49–59.89, *p* < 0.001).

Moreover, both the anterior location of the tumor (RR: 1.54, 95%CI: 0.44–3.48, *p* < 0.001) and a positive mCRM (RR: 2.78, 95%CI: 1.09–7.05, *p* = 0.03) had a significant impact on the discovery of a pCRM in postsurgical pathological examinations. On the other hand, a distance of less than 4 cm did not significantly impact the evaluated outcome (*p* = 609).

In the third stage of our analysis, we performed a sensitivity analysis that included MRI parameters taken individually or combined as predictors and the pCRM as the outcome. Our results are presented in Table 3.

When individually evaluated, the best MRI predictor for the detection of a pCRM in the postsurgical histopathological examination is mrEMVI, which achieved a sensitivity (Se) of 77.78%, a specificity (Sp) of 87.38%, a negative predictive value (NPV) of 97.83%, and an accuracy of 86.61%.

This MRI predictor was followed by a positive mCRM, which achieved an Se of 66.67%, an Sp of 80.58%, an NPV of 96.51%, and an accuracy of 79.46%. On the other hand, a distance of less than 4 cm from the anal verge achieved modest results in terms of predictive power, with an accuracy of 61.61%.

The combined models were characterized by a high negative predictive value (over 90%), but with the cost of reduced sensitivity compared with individual MRI markers. The best predictive performance was achieved by a model which comprised all MRI predictors (mCRM+ mrEMVI+ anterior location+ < 4 cm from anal verge), with an Se of 66.67%, Sp of 88.46%, NPV of 96.84%, and accuracy of 86.73%.

**Figure 3:**
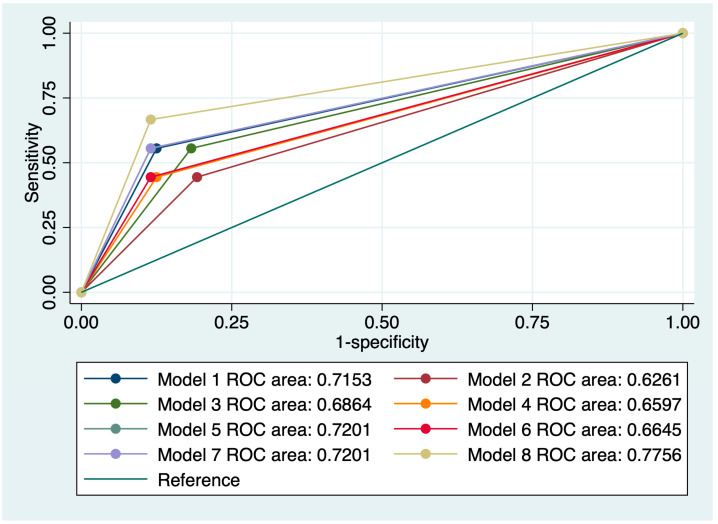
Comparison of ROC curves corresponding to 8 models used for the prediction of pCRM positivity.

A comparison of the models considering the value of the area under the curve (AUC) is presented in Figure 3. When evaluating the MRI predictors individually, the best AUC value was obtained for mrEMVI (0.825). A model which comprised all MRI predictors (mCRM+ mrEMVI+ anterior location+ < 4 cm from anal verge) achieved the highest AUC value from all combined models (0.775).

**Figure 4:**
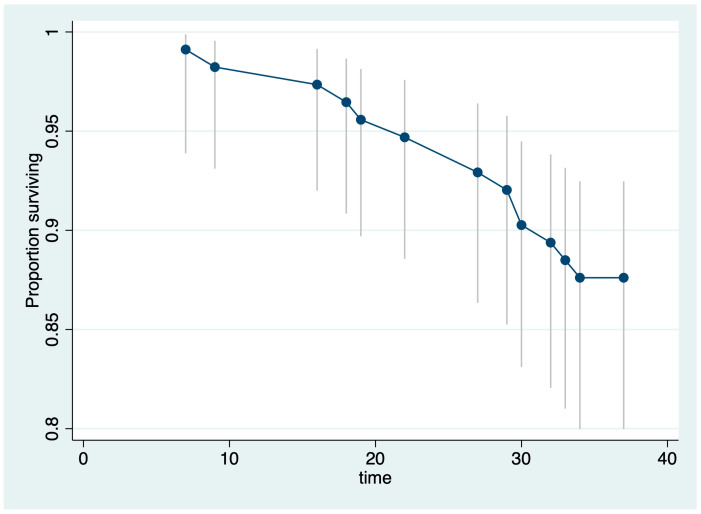
Life plot for aggregate survival data and confidence intervals.

**Figure 5:**
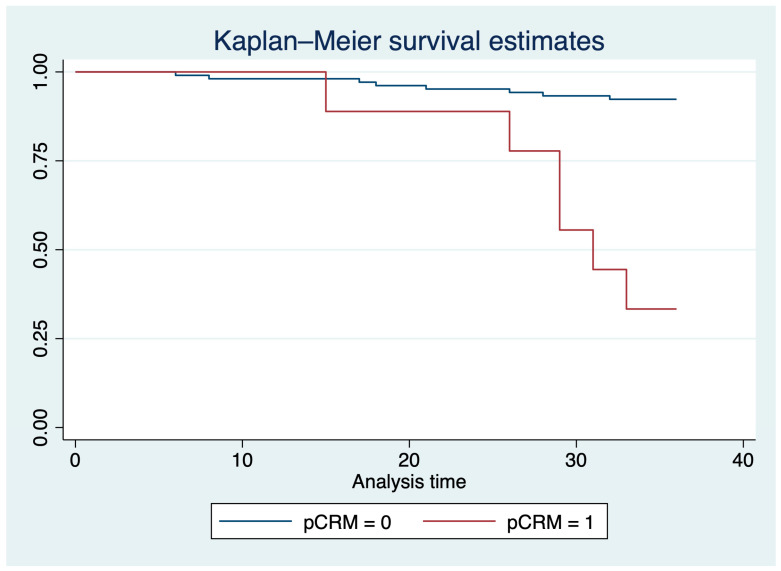
Kaplan–Meier survival estimates based on the presence or absence of a positive pCRM result in postsurgical evaluation.

**Figure 6:**
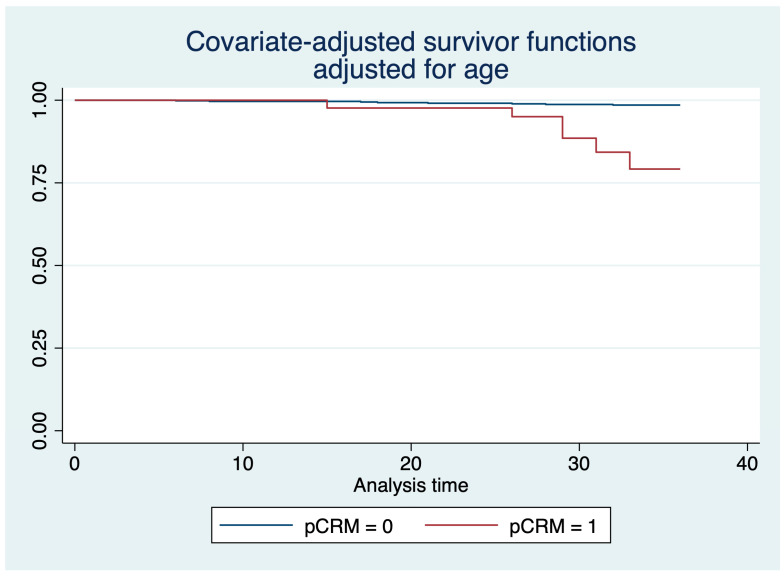
Kaplan–Meier survival estimates based on the presence or absence of a positive pCRM result in postsurgical evaluation and adjusted for age.

A total of 14 (12.39%) patients died during follow-up, and their death was related to the oncologic diagnosis. From the total deaths, six were recorded in the positive pCRM group (42.86%), and eight in the negative pCRM group (57.14%). A graphical representation of the proportion of patients surviving during the 36-month follow-up, along with their confidence intervals, is presented in Figure 4. The Kaplan–Meier survival estimates, segregated based on groups or adjusted for age, are presented in Figure 5 and Figure 6.

There was a statistically significant difference between groups regarding the mortality rate in a 36-month interval, even after adjustment with the age covariate (*p* < 0.001).

## 4. Discussion

A positive pathological circumferential resection margin plays a crucial role in rectal cancer surgery as it serves as a significant prognostic factor, impacting both local recurrence and overall survival rates. A review conducted by Nagtegaal et al. has shown that a positive pCRM after neoadjuvant therapy is a powerful predictor of the development of distant metastases from the primary rectal tumor (HR = 2.8; 95% CI, 1.9 to 4.3) and survival in patients with rectal tumors who underwent various surgical procedures (HR = 1.7; 95% CI, 1.3 to 2.3) [21], thus outlining the need to obtain clean margins after surgical procedures.

The objective of this prospective study was to assess the predictive accuracy of different MRI parameters, either individually or in combination, for predicting pCRM in patients diagnosed with rectal cancer. Our results indicated that mrEMVI achieved the best results for the detection of a pCRM in the postsurgical histopathological examination, with an accuracy of 86.61% and an AUC value of 0.825. In our study, EMVI was positive for seven patients (77.78%).

These results are partially in line with previously published data. For example, Smith et al. discovered a significant association between EMVI-positive tumors and positive CRM (*p* < 0.013) [22], and the MERCURY II trial indicated that mrEMVI had a significant correlation with a 3.8-fold higher likelihood of pCRM involvement [11]. On the other hand, a retrospective study conducted by Patra et al. showed that the only predictor associated with a positive pCRM was a positive CRM on MRI (*p* = 0.01) [23]. However, the same study indicated a higher frequency of mrEMVI and of the anterior location of the rectal tumors in patients who had a positive pCRM [23].

Kim et al. performed a retrospective study that evaluated the diagnostic accuracy of positive mrEMVI for tumor deposits on pathological samples, and its association with the prognosis of patients with locally advanced rectal cancer after neoadjuvant therapy [24]. Their results showed that a positive mrEMVI had an Se of 62% and an Sp of 93% for the prediction of the evaluated outcome. Moreover, the same study demonstrated that the presence of mrEMVI was associated with worse disease-free survival and overall survival.

A recent meta-analysis indicated an almost four-fold increase in the risk of developing metastases in patients with rectal cancer and positive mrEMVI [25]. Other studies outlined the association between a positive EMVI with distant metastasis of rectal cancer [26,27], thus outlining the need for careful evaluation of this imaging marker after neoadjuvant therapy.

Some studies have outlined a statistically significant association between a positive pCRM and the anterior locations of rectal tumors. For example, Mo et al. discovered that there was a higher occurrence of pCRM positivity in anterior tumors compared to non-anterior tumors (*p* < 0.007). Also, the MERCYRY II study revealed a significant 2.8-fold increase in pCRM involvement among patients with rectal tumors and an anterior quadrant invasion [11]. In our study, the anterior location of the rectal tumors was significantly associated with positive pCRM, but it had a low sensitivity (44.44%), and relatively high specificity (80.58%) and NPV (94.32%), with a good accuracy (77.68%) when it was individually assessed. Thus, our findings confirm the literature data regarding this predictor.

On the other hand, a distance lower than 4 cm from the tumor to the anal verge as detected by MRI examination was not statistically associated with a positive pCRM in our cohort of patients. Moreover, this individual predictor achieved modest predictive performance for the evaluated outcome, with an Se of 66.67%, Sp of 61.17%, accuracy of 61.61%, and AUC value of 0.639.

The literature provides varying descriptions of the impact and association of this parameter with the detection of a positive pCRM. For example, Patra et al. did not find a significant difference in pCRM involvement for a distance cut-off of less than 4 cm from the anal verge [23], while Khan et al. outlined significantly higher rates of positive pCRM when this distance was less than 5 cm [28].

When we evaluated the predictive performance of combined models that included MRI predictors for the prediction of positive pCRM, we observed that all models were characterized by a high negative predictive value (over 90%), but with the cost of reduced sensitivity compared with individual MRI markers. Moreover, our results indicated that the model which comprised all MRI predictors achieved the best predictive performance for the evaluated outcome, with an accuracy of 86.73% and an AUC value of 0.775.

Several models for the prediction of a positive pCRM have been proposed in the literature. Ju et al. conducted a retrospective multicentric study on 275 patients with rectal cancers who underwent neoadjuvant therapy, and they investigated the predictive performance of a radiomics prediction model for predicting perioperative surgical margins [29]. This model included both MRI predictors and clinical features, and it achieved an AUC value of 0.848 in the validation stage. This could potentially indicate the need to include clinical characteristics in the combined models for the prediction of positive pCRM.

We did not include clinical features in our model because the only significant clinical parameter between groups was the mean age of the patients. This parameter was significantly higher for the group of patients with positive circumferential resection margin determined in histopathological examination (*p* = 0.03). Also, other patient characteristics such as gender, medium of living, body mass index (BMI), and smoking status did not significantly differ between groups (*p* > 0.05).

Previous studies have outlined the association between advanced age and the more frequent detection of a positive circumferential resection margin in patients with rectal adenocarcinomas [30], although this association is controversial [31,32].

Another model for the prediction of positive pCRM was proposed in a prospective study by Roodbeen et al. on a cohort of patients with rectal cancer that underwent transanal total mesorectal excision, and it included tumors located up to 1 cm from the anal verge, anterior tumors, cT4 tumors, positive EMVI, and threatened or involved CRM on presurgical MRI [33]. This model had an AUC value of 0.715, and their results are comparable with those achieved by our combined model.

When managing low rectal adenocarcinoma, the goal is to provide effective oncological treatment while also preserving the patient’s quality of life. Sphincter-sparing surgery becomes a favorable option when there is no evidence of tumor invasion into the intersphincteric space [34]. This approach can help to avert the physical and psychological burdens associated with rectal amputation, such as permanent colostomy [35]. Patients who qualify for sphincter-sparing techniques are offered a less invalidating surgical option and the possibility of maintaining continence.

The decision to pursue sphincter-sparing surgery is critically dependent on meticulous preoperative staging, an area where high-resolution MRI plays a pivotal role. Accurate MRI staging can discern the precise extent of the tumor, assess the involvement of the mesorectal fascia, and evaluate the condition of the intersphincteric space [36]. A correct MRI assessment helps in delineating an optimal surgical plan that aims to manage the adenocarcinoma effectively while sparing the anal sphincters, providing the patient with an opportunity for a better postoperative quality of life [37].

A recent study by Zhu et al. evaluated the accuracy of several MRI parameters in predicting the feasibility of sphincter-sparing surgery in patients with low or middle rectal cancer [38]. The authors demonstrated that the best predictor for the evaluated outcome was the distance from the lower edge of the tumor to the upper margin of the internal sphincter, which achieved an AUC value of 0.997 for a cut-off value of 2 cm in the training phase as well as an AUC value of 0.996 in the validation phase, with an overall accuracy of 99.1%. The importance of precise RMN staging cannot be overstated, as it guides the surgical approach, ensuring that oncological safety is not compromised when electing for a more conservative surgical option.

In the treatment of rectal cancer, a “wait-and-see” approach following neoadjuvant therapy has emerged as a potential strategy for select patients displaying complete clinical response [39]. This shift towards a conservative management strategy, where traditional surgery is deferred, underscores the need for rigorous patient follow-up. In this context, MRI, with its detailed soft-tissue contrast resolution, allows clinicians to monitor the tumor bed for any signs of residual disease or recurrence with a high degree of precision [40].

Artificial intelligence and risk stratification have been gaining more interest in the field of predictive medicine, and they have been frequently used in imaging data modeling in recent years [41,42,43,44,45,46]. Moreover, the correct reporting of positive circumferential margin on MRI has been the subject of debate. One study conducted by Dongsheng et al. investigated the predictive performance of a convolutional neural network for the prediction of positive CRM in patients with rectal cancer on MRI images [47]. The results from this study indicated a good predictive performance, with an Se of 83.8%, an Sp of 95.6%, and an accuracy of 93.2%.

Additionally, another convolutional neural network (faster regional convolutional neural network) was employed by Xu and colleagues for the prediction of positive pCRM using MRI images of patients with rectal cancer [48]. The authors demonstrated that this type of neural network was able to predict a positive pCRM with an accuracy of 88.4%, an Se of 85.7%, an Sp of 89.8%, and an AUC value of 0.934.

A positive pCRM was associated with worse recurrence-free survival, non-local recurrence-free survival, and cancer-specific survival as demonstrated in numerous observational studies [20,49,50].

Moreover, a recent systematic review and meta-analysis of 75 studies that included patients with rectal cancer concluded a positive circumferential resection margin is an independent prognostic factor for local recurrence and survival [51]. Our results indicated that the survival rates were significantly lower in patients with positive pCRM after surgical interventions for rectal cancer, thus confirming the literature data.

The results from this study should be interpreted considering the following limitations: the small cohort of patients, the limited time-frame for patients’ follow-up, and the small number of MRI parameters evaluated. On the other hand, this study has the advantage of a prospective design and a homogeneous group of patients considering the rectal tumor type and demographic data.

We hypothesize that further studies, on a larger cohort of patients, could include individual or combined MRI parameters in several machine learning or convolutional neural networks in order to better establish their predictive performance. These approaches allow better image segmentation or feature discrimination and allow the analysis of a large dataset, even with high rates of missing data [52,53]. Moreover, the combined models could help us identify those patients with positive pCRM who are at higher risk of developing local recurrence or distant metastasis, after validation at our local institution and in larger cohorts of patients. This type of risk stratification would help in the planning of the best surgical approach and follow-up plan.

## 5. Conclusions

A positive pathological circumferential resection margin is an important prognostic factor for the survival of patients with rectal cancer, and the preoperative prediction of this marker should be improved for better patient management.

In this prospective study, mrEMVI achieved the best individual results for the detection of a pCRM in the postsurgical histopathological examination, while the best predictive performance for the prediction of this outcome was achieved by a combined model that comprised mCRM, mrEMVI, the anterior location of the tumor, and a distance of less than 4 cm from the anal verge.

Combined models could be incorporated in the presurgical evaluation of patients with low rectal adenocarcinoma for the risk stratification of those patients who are at high risk of local recurrence or distant metastasis due to a positive pCRM.

## Figures and Tables

**Table 1:** Demographic and clinical characteristics segregated considering the second histopathological examination of the cervical probes.

Variable	pCRM Positive (Group 1, n = 9 Patients)	pCRM Negative (Group 2, n = 103 Patients)	*p* Value
Age, years (mean ± SD)	68.61 ± 9.45	61.33 ± 12.30	0.03
Gender (n/%)	Male = 6 (66.67%) Female = 3 (33.33%)	Male = 64 (62.14%) Female = 39 (37.86%)	0.07
Medium (n/%)	Urban = 5 (55.5%) Rural = 4 (44.4%)	Urban = 42 (40.78%) Rural = 61 (59.2%)	0.74
BMI, kg/m^2^ (mean ± SD)	24.10 ± 3.82	24.14 ± 3.9	0.89
Smoking (n/%)	Yes = 2 (22.22%)	Yes = 15 (14.56%)	0.53
Positive mCRM (n/%)	Yes = 6 (66.67%)	Yes = 20 (19.42%)	0.001
Positive EMVI (n/%)	Yes = 7 (77.78%)	Yes = 13 (12.62%)	<0.001
Location (n/%)	Anterior = 5 (55.56%) Other = 4 (44.44%)	Anterior = 20 (19.42%) Other = 83 (80.58%)	0.01
Distance from anal verge (n/%)	<4 cm = 5 (55.56%) >4 cm = 4 (44.44%)	<4 cm = 40 (38.83%) >4 cm = 63 (61.17%)	0.32

Table legend: pCRM—pathological (positive) circumferential resection margin; mCRM—positive circumferential resection margin on MRI; mrEMVI—extramural venous invasion on MRI; SD—standard deviation; BMI—body mass index.

**Table 2:** Results from the generalized linear model for evaluating the impact of MRI predictors on the pCRM positivity.

Predictor	RR	Standard Error	95% CI (Lower Bound and Upper Bound)	*p* Value
mCRM	2.78	1.32	1.09–7.05	0.03
mrEMVI	40.97	28.46	10.49–59.89	<0.001
Anterior location	1.54	0.46	0.44–3.48	<0.001
Less than 4 cm from anal verge	0.78	0.37	0.30–1.98	0.609

Table legend: RR—risk ratio; CI—confidence interval; mCRM—positive circumferential resection margin on MRI; mrEMVI—extramural venous invasion on MRI.

**Table 3:** Results from the sensitivity analysis for evaluating the predictive performance of individual or combined MRI predictors on the pCRM positivity.

Index Test	Se (%)	SP (%)	NPV (%)	AUC	Accuracy
mCRM	66.67	80.58	96.51	0.736	79.46
mrEMVI	77.78	87.38	97.83	0.825	86.61
Anterior location	44.44	80.58	94.32	0.625	77.68
Less than 4 cm from anal verge	66.67	61.17	95.45	0.639	61.61
mCRM+ mrEMVI (model 1)	55.56	87.50	95.79	0.7153	84.96
mCRM+ anterior location (model 2)	44.44	80.77	94.38	0.626	77.88
mCRM+ < 4 cm from anal verge (model 3)	55.56	81.73	95.51	0.686	79.65
mrEMVI+ anterior location (model 4)	44.44	87.50	94.79	0.659	84.07
mrEMVI+ < 4 cm from anal verge (model 5)	55.56	88.46	95.83	0.720	85.84
mCRM+ mrEMVI+ anterior location (model 6)	44.44	88.46	94.85	0.664	84.96
mrEMVI+ anterior location+ < 4 cm from anal verge (model 7)	55.56	88.46	95.83	0.720	85.84
mCRM+ mrEMVI+ anterior location+ < 4 cm from anal verge (model 8)	66.67	88.46	96.84	0.775	86.73

Table legend: Se—sensitivity; Sp—specificity; NPV—negative predictive value; AUC—area under the curve; mCRM—positive circumferential resection margin on MRI; mrEMVI—extramural venous invasion on MRI.

## Data Availability

The datasets are available from the corresponding authors upon a reasonable request due to local policies.
